# Grounds, Beds, and Reefs of the Endangered Coral *Cladocora caespitosa* With Description of the Tremiti Reef

**DOI:** 10.1002/ece3.72871

**Published:** 2026-02-24

**Authors:** Giovanni Chimienti, Andrea Tursi, Alessia Logrieco, Francesco De Giosa, Francesco Mastrototaro

**Affiliations:** ^1^ Department of Biosciences, Biotechnology, and Environment University of Bari Aldo Moro Bari Italy; ^2^ CoNISMa National Interuniversity Consortium for Marine Sciences Rome Italy; ^3^ Department of Earth and Geo‐Environmental Sciences University of Bari Aldo Moro Bari Italy; ^4^ Environmental Surveys S.r.l. (ENSU) Taranto Italy

**Keywords:** biogeography, habitat mapping, hermatypic, marine protected area, Mediterranean Sea, Scleractinia

## Abstract

*Cladocora caespitosa* (Cnidaria: Scleractinia: Cladocoridae) is an endangered coral species that forms peculiar ecosystems in the Mediterranean Sea. These are *Cladocora* grounds (aggregations of colonies, more or less spaced, on rocky bottoms), *Cladocora* beds (aggregations of unattached colonies, more or less spaced, on both soft and hard bottoms), and *Cladocora* reefs (formations with large colonies, mostly in contact with each other or with little space between them). This study provides an overview of the known presence and distribution of these three ecosystem types and describes newly discovered ones at Tremiti Islands Marine Protected Area (Adriatic Sea, Italy). Here, the *Cladocora* ground was present between 5 and 10 m depth, extending on 25,687 m^2^ (2.57 ha) with a mean density of 4.58 ± 0.45 colonies m^−2^, the *Cladocora* bed occurred between 15 and 23 m depth over 47,120 m^2^ (4.71 ha), with 8.59 ± 1.46 colonies m^−2^, while the *Cladocora* reef developed at 25–35 m depth, with a patchy distribution over 14,364 m^2^ (1.43 ha) and a mean density of 3.89 ± 0.17 colonies m^−2^. The spatial distribution, biometry, size structure, and conservation status of the *Cladocora* formations have been investigated and compared at the Mediterranean scale, where a total of 40 grounds, 3 beds, and 13 reefs have been currently documented. The role of environmental forcings in shaping the population structure of *C. caespitosa* and possibly driving the occurrence of each ecosystem type is discussed.

## Graphical abstract

This study provides an overview of the distribution of the three ecosystem types formed by *Cladocora caespitosa* and describes newly discovered ones at Tremiti Islands (Adriatic Sea, Italy). The spatial distribution, biometry, size structure, and conservation status of the Cladocora formations have been investigated and compared at the Mediterranean scale, where a total of 40 grounds, 3 beds, and 13 reefs have been currently documented.

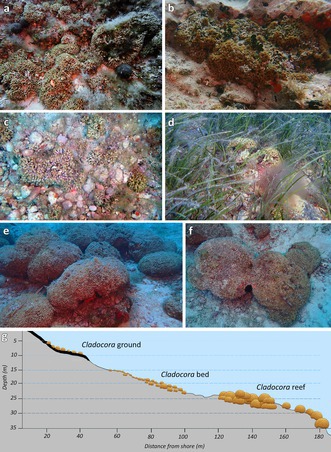

## 1 Introduction

The pillow coral *Cladocora caespitosa* (Linnaeus 1767) is a zooxanthellate, hermatypic coral endemic to the Mediterranean Sea. This scleractinian can be found within a wide variety of conditions, from 1 to approximately 40 m depth within both protected and exposed areas, under low‐light and well‐lit conditions, on soft and hard bottoms, and in oligotrophic to eutrophic waters (Schiller 1993; Morri et al. 1994; Bitar and Zibrowius 1997; Peirano et al. 2005; Rodolfo‐Metalpa et al. 2006; Kružić and Benković 2008; Kersting and Linares 2012; Chefaoui et al. 2017). The adaptation of *C. caespitosa* to live in a wide variety of habitats stands in its ability to rely on heterotrophy in low light conditions (Hoogenboom et al. 2010; Ferrier‐Pagès et al. 2013), to produce allelochemicals to compete with macroalgae and avoid epibiosis (Kersting et al. 2014), and to modify the morphology of its colonies under different hydrodynamic regimes and seabed types (Kružić and Benković 2008; Kersting and Linares 2012; Chimienti et al. 2025). Extensive *C. caespitosa* carbonate frameworks were more common during the Miocene (Dabrio et al. 1981; Pomar 1991), the Pliocene (Aguirre and Jiménez 1998), and the Pleistocene (Bernasconi et al. 1997) than in the present time, most of the known populations nowadays consisting of small, interspersed colonies with a patchy distribution. Especially in the last few decades, this species has experienced an overall decline mostly due to global warming and heat waves, which triggered mass mortality events at the basin scale (Cerrano et al. 2000; Perez et al. 2000; Rodolfo‐Metalpa et al. 2005; Kružić et al. 2012, 2025; Kersting et al. 2013; Jiménez et al. 2016; Rubio‐Portillo et al. 2016; Garrabou et al. 2022). Other human‐related disturbances, such as the spread of alien species (Kružić et al. 2008; Kersting et al. 2014, 2015), the eutrophication (Kružić and Požar‐Domac 2007), the marine pollution (El Kateb et al. 2016), and the coastal development (Casado‐Amezúa et al. 2015), also affect the conservation status of *C. caespitosa* . In fact, the International Union for the Conservation of Nature (IUCN) classified this species as endangered (Otero et al. 2017).

Large aggregations of *C. caespitosa* can occur with various densities and characteristics, forming different types of ecosystems, sometimes mixed. The nomenclature of the *C. caespitosa* formations has been recently revised, distinguishing between reefs (also known as banks), grounds, and corallith beds (Chimienti et al. 2025). Numerous, spaced colonies attached to a rocky substrate form a *Cladocora* ground (e.g., Kersting et al. 2023). *Cladocora* beds are constituted of dense populations living as unattached coral nodules, known as coralliths, on soft (Laborel 1961; Chimienti et al. 2025; Kružić et al. 2025) or hard bottoms (Kersting et al. 2017a); coralliths are initially spherical with living polyps at 360° due to rollover, while they eventually stop rolling when reaching a certain size and develop as hemispherical colonies. Numerous colonies, often coalescing, in contact or with little space between them, create large carbonate frameworks called *Cladocora* reefs (e.g., Peirano et al. 1994; Kružić and Benković 2008). Reefs may grow from grounds (on hard bottoms) and beds (on soft bottoms) under conditions of undisturbed accretion (Laborel 1961; Peirano et al. 1994), and beds may derive from reefs and grounds, possibly through colony fragmentation or recruitment (Kersting et al. 2017b; Kružić et al. 2025). Nowadays, *C. caespitosa* ecosystems are considered rare in the basin, with reefs and corallith beds being very rare (Chefaoui et al. 2017; Ingrosso et al. 2018; Chimienti et al. 2025). Comprehensive descriptions of grounds, beds, and reefs remain very limited, and their spatial extent, structure, and conservation status are still poorly understood.

In this study, we aimed at mapping the distribution of a dense and peculiar *C. caespitosa* bed recently found at Tremiti Islands Marine Protected Area (MPA), in the Southern Adriatic Sea, Italy (Chimienti et al. 2025). A combination of mapping and diving surveys, however, allowed the discovery of a *C. caespitosa* ground in shallower waters compared to the corallith bed, and a *C. caespitosa* reef in deeper waters. This combination of ground, corallith bed, and reef occurring within the same area has not previously been documented at any other Mediterranean site, and the Tremiti Islands therefore provide a unique natural setting to investigate how local environmental conditions shape the development and persistence of distinct *C. caespitosa* formations. These ecosystems are here mapped and characterized for the first time through an integrated approach combining high‐resolution acoustic surveys and photographic sampling via SCUBA diving, allowing us to quantify their spatial extent, colony density, cover, morphometry, size structure, and conservation status. In addition, we provide an updated and revised synthesis of all known *C. caespitosa* grounds, beds, and reefs across the Mediterranean Sea, enabling a comparative assessment of their biogeography, structural traits, and rarity across the basin.

## 2 Materials and Methods

### 2.1 Habitat Mapping

The study was carried out at Tremiti Islands MPA, along the Italian coast of the Southern Adriatic Sea, in both the Zone B and C of the MPA, such as the general reserve and the buffer zone, respectively. Details are provided in Chimienti, De Padova, et al. (2020); Chimienti et al. (2025) and Tursi et al. (2022). Mapping was carried out during 2023 using a Klein3000 Side‐Scan Sonar, for a total of ca. 25 km^2^ mapped. Data were acquired with a double frequency of 100 and 500 kHz, simultaneously, using a swath width of 100 m, with 50% of overlap between adjacent lines. Side‐Scan Sonar data were processed using the CARIS HIPS software, according to the following steps: data conversion; navigation processing; slant range correction; time varying gain application; attitude data editing; data mosaicing; data output. Geo‐referenced, gray‐tone acoustic images of the seafloor with a 0.2 m cell resolution were produced. Ground, bed, and reef ecosystems formed by *C. caespitosa* were identified by combining acoustic data with visual ground truthing by scuba diving and, on shallow rocky bottoms, based on direct underwater surveys using a georeferenced diver propulsion vehicle.

### 2.2 Visual Surveys

The site of *Grotta del Sale* (GDS; 42.10791° N, 15.48938° E) was selected as the main study site for the visual surveys due to the presence of the three *Cladocora* ecosystem types. These were studied by SCUBA diving through photographic surveys during July 2023. Underwater photos were taken using an Olympus TG‐7 camera equipped with lights and two laser beams for size reference (distance: 10 cm). The camera was positioned at a 90° angle with the coral colonies to obtain a top‐down perspective, each photo not overlapping with the others and representing a sampling unit.

The *Cladocora* ground was surveyed over 108 m^2^, from 5 to 10 m depth, with 131 sampling units. The *Cladocora* bed was investigated over 143 m^2^, from 20 to 22 m depth, with 172 sampling units. The *Cladocora* reef was surveyed over 150 m^2^, from 25 to 30 m depth, with 180 sampling units. Images were processed using the photoQuad 1.4 software to assess: (i) colony density and cover on the sea bottom, (ii) biometric parameters, and (iii) the conservation status of each colony. Colonies in advanced coalescence stages, so that it was not possible to clearly distinguish their borders, were considered as a single colony. Colonies at early coalescence stages were considered as different colonies.

A square sampling unit of 0.8 m^2^ was fitted in each image imported into the photoQuad 1.4 environment. Coral density (colonies m^−2^) and coral cover (% of the surface occupied by all the colonies within the sampling unit) were assessed for each sampling unit. Both coral density and cover for each ecosystem type were calculated considering all sampling units and expressed as mean ± standard error. Both major (D1c, cm) and minor (D2c, cm) colony axes were calculated for each colony and expressed at the population level as mean ± standard error. Both major (D1p, cm) and minor (D2p, cm) axes of 10 random polyps of each colony were also measured for 100 colonies within each ecosystem type and expressed as mean ± standard error at both colony and population level. The *C. caespitosa* population size structure was assessed based on D1c, considering size classes of 5 cm (*sensu* Zunino et al. 2018; Chimienti et al. 2025). Size frequency, skewness, and kurtosis were calculated using R software (v. 4.3.2).

The conservation status was evaluated for each *C. caespitosa* colony, considering three categories: healthy (polyps alive, brownish green in color, without signs of damage, disease, necrosis, or epibiosis); dead (necrotic or dead polyps); and epibionted (presence of benthic organisms on the coral colony). Since no formal or standardized scale exists for evaluating the conservation status of *C. caespitosa* ecosystems, our classification followed descriptive criteria used in studies assessing colony health, necrosis, and epibiosis in Mediterranean scleractinians (e.g., Rodolfo‐Metalpa et al. 2005; Kersting et al. 2013; Jiménez et al. 2016). Importantly, colonies were not assigned to an overall qualitative condition class, but the three descriptors (healthy surface, necrosis, and epibiosis) were quantified independently as continuous variables, allowing each colony to be evaluated based on the proportion of its living, necrotic, or epibiont‐covered tissue. Each colony was therefore categorized based on the percentage of healthy, dead, and epibionted surface. Bleaching was negligible, limited to a few colonies (< 2%), each having a few polyps bleached (0.01%–1% of colony area); thus, it was excluded from the analysis. Each descriptor was quantified (cm^2^) and expressed as percentage cover compared to the colony area, then expressed for each *Cladocora* ecosystem as mean ± standard error. Epibionts were identified at the lowest taxonomic level from the images or through qualitative sampling. Macroalgae were identified based on morpho‐anatomical features of their thalli and through microscopical sections. The contribution of each epibiont in terms of cover was expressed as a percentage with respect to the total epibionted colony surface for each ecosystem type, calculated as the sum of the epibionted area of each colony.

### 2.3 Environmental Parameters

Water temperature was recorded through fixed underwater thermometer stations placed every 5 m depth (from 5 to 40 m) in two sites at the north and south tips of the archipelago. Temperature was recorded continuously (1‐h intervals) from 1 January to 31 December 2023. Temperature profiles along the water column for the whole year were obtained using the OceanDataView software (Schlitzer 2022).

### 2.4 Statistical Analysis

All statistical analyses were performed in R environment (v. 4.3.2). To investigate the relationships among *C. caespitosa* colony size metrics, bivariate correlation analyses were carried out between morphometric parameters (i.e., D1c, D2c, colony area, D1p, D2p), as well as between the descriptors of the conservation status (i.e., percent of healthy surface, mortality, epibiosis). Before correlation, data normality was assessed using the Shapiro–Wilk test. Based on the outcome, Pearson's correlation coefficient was applied to normally distributed data, while Spearman's rank correlation was used for non‐normally distributed variables. Results were visualized through scatterplots with linear regression lines, 95% confidence intervals, and reported as correlation coefficients (*R*) along with associated *p*‐values.

To evaluate whether colony density and percentage cover varied across the sampling grid, we applied a one‐sample Kolmogorov–Smirnov (K–S) test to the distribution of SU‐level values. In both cases, the empirical cumulative distribution of the sampling‐unit measurements was compared with a theoretical uniform distribution, which represents the expectation that all sampling units host similar values. The K–S test was used exclusively to assess whether density or cover values at the sampling‐unit level were homogeneous or heterogeneous across the grid. A significant deviation from the uniform model (*p* < 0.05) indicated heterogeneity among sampling units, whereas a non‐significant result suggested that no detectable departure from homogeneity was present at this scale. Because the imagery provides SU‐aggregated data rather than the coordinates of individual colonies, this approach does not constitute a spatial point‐pattern analysis and cannot resolve geometric aggregation of colonies in two‐dimensional space, while it quantifies mesoscale spatial heterogeneity at the resolution of the sampling units.

To compare morphometric traits and conservation status across the three *Cladocora* ecosystem types, a non‐parametric Kruskal–Wallis test was applied following the D'Agostino‐Pearson normality test, followed by pairwise post hoc comparisons using Dunn's test with Bonferroni correction. Group differences were visualized using boxplots with jittered raw data and significance letters indicating statistically distinct groups (*p* < 0.05).

## 3 Results

### 3.1 Spatial and Bathymetric Distribution of *Cladocora* Ecosystems at Tremiti Islands

**FIGURE 1:**
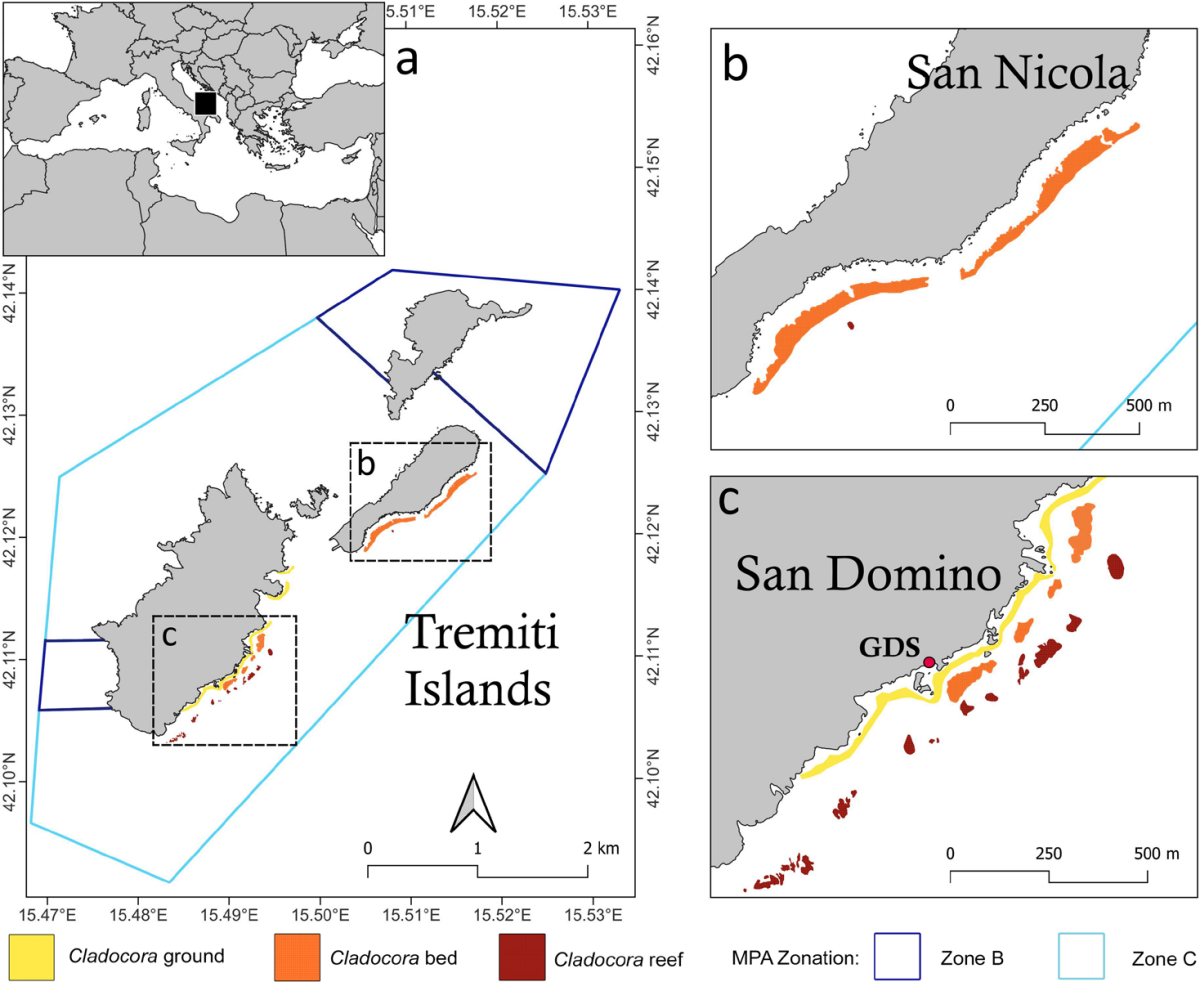
Distribution of *Cladocora* ecosystems at (a) Tremiti Islands Marine Protected Area (MPA), with indications of the three ecosystem types and the MPA zonation (Zone B, general reserve; Zone C, buffer zone). (b) Focus on San Nicola Island; and (c) focus on San Domino Island.

**FIGURE 2:**
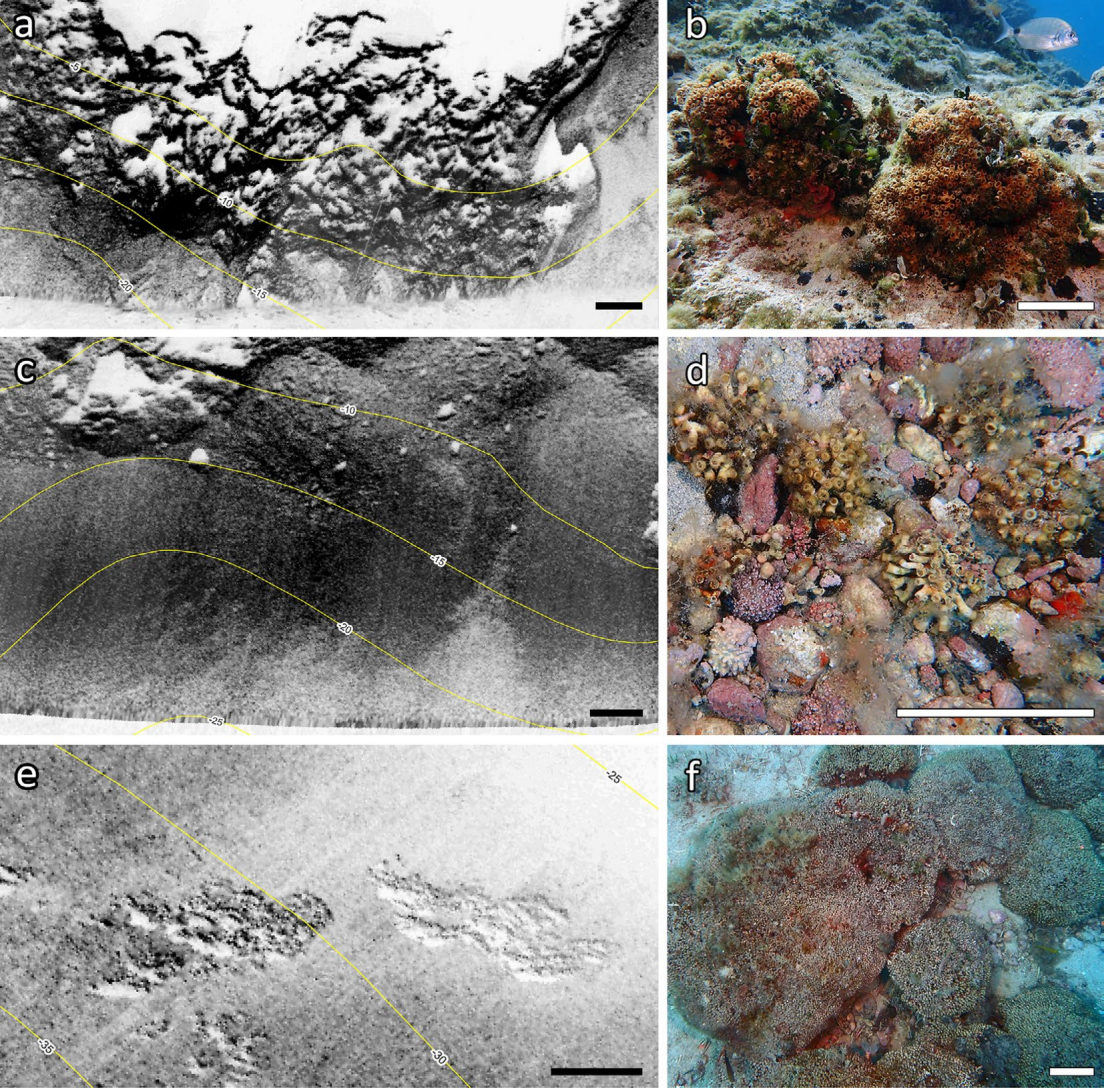
Mapping of *Cladocora* ecosystems at Tremiti Islands, with acoustic response (left, with 5‐m isobaths) and visual ground truthing (right). (a) Sonogram of the rocky bottom where it is not possible to detect the presence of the *Cladocora* ground; (b) example of the ground; (c) sonogram where the dark area corresponds to the *Cladocora* bed, while deeper that light area indicates a sandy/detritic bottom without *C. caespitosa* ; (d) example of the corallith bed; (e) sonogram where it is evident a patch of *Cladocora* reef (structure on the right) and a patch of the coralligenous (dark structure on the left) among a sandy seabed; (f) example of reef. Scale bars: a,c,e: 10 m; b,d,f: 10 cm.

**FIGURE 3:**
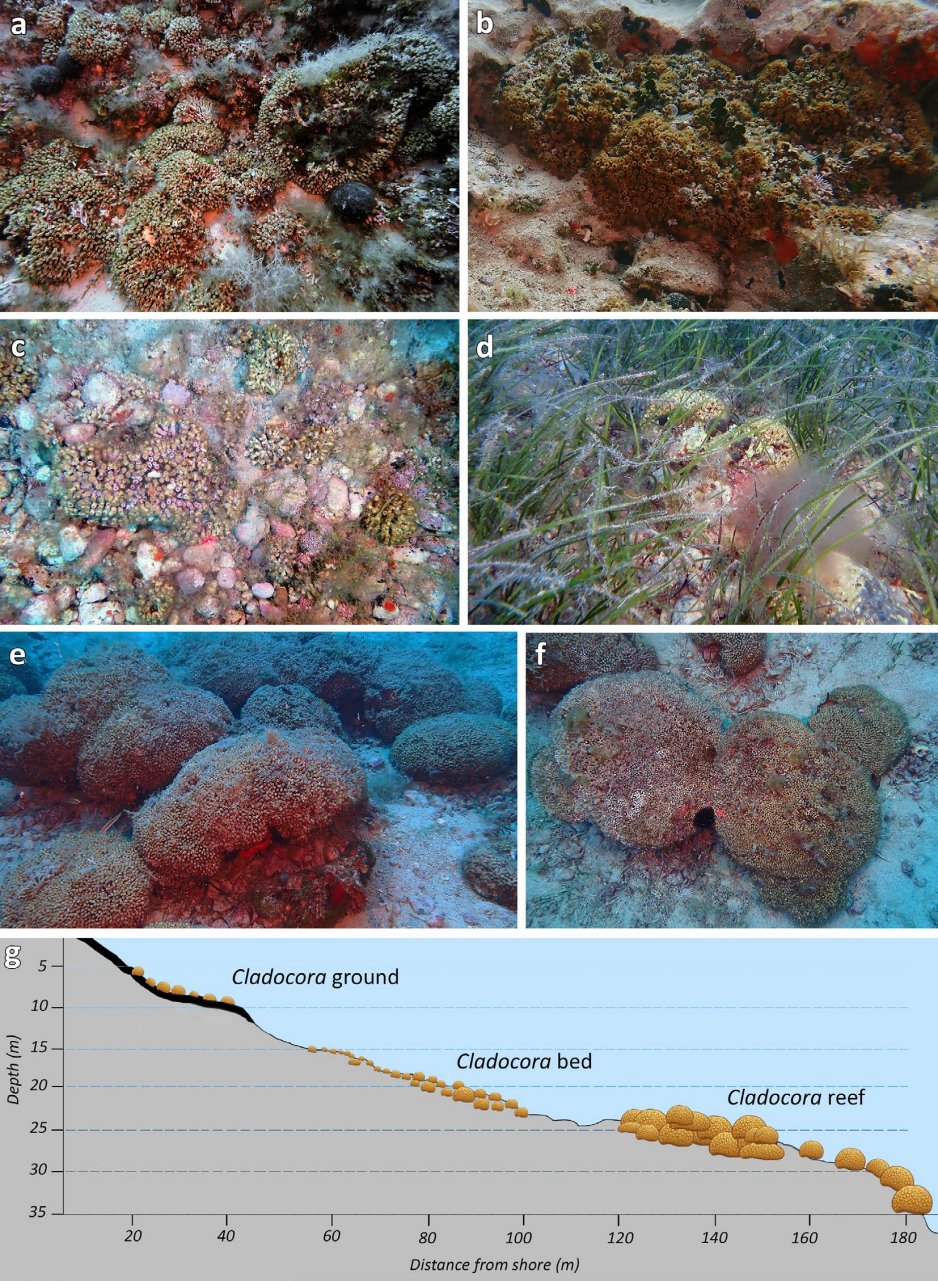
*Cladocora* ecosystems. (a) ground among algae; (b) ground on barren; (c) corallith bed with rhodolith bed; (d) bed with *Cymodocea nodosa*; (e) reef; (f) coalescence of at least six colonies; (g) schematic distribution of the three *Cladocora* ecosystems at Tremiti Islands with indication of hard (thick line) and soft (thin line) substrates.

From 5 to 35 m depth, different *Cladocora* ecosystems were detected in the study area, within Zone C of the MPA (Figure 1a–c). Each ecosystem type occurred in a distinct depth range, with no overlap among formations. The *Cladocora* ground was observed between 5 and 10 m depth, on hard bottoms with different inclinations, covering an overall surface of 25,687 m^2^ (2.57 ha) within the Tremiti archipelago. Due to its occurrence on a complex rocky bottom, the *Cladocora* ground was indistinguishable from other seabed biological features (e.g., barren, association of photophilous algae) using acoustic mapping (Figure 2a,b), the only effective mapping technique being georeferenced visual surveys. The *Cladocora* ground was present on barren rocky habitats (Figure 3b), mixed with *P. oceanica* , as well as among algal associations with Chlorophyta (i.e., genera *Codium*, *Cladophora*, *Pseudochrolodesmis*), Rhodophyta (i.e., genera *Amphiroa*, *Ellissolandia*, *Laurencia*, *Lithophyllum*, *Peyssonnelia*), and Ochrophyta (i.e., genera *Dictyota*, *Padina*) (Figure 3a). Rocky bottoms below 10 m depth were not particularly colonized by *C. caespitosa* . From 15 to 23 m depth, an extensive corallith bed was identified over a total area of 42,064 m^2^ (4.21 ha). The bed was mostly found in association with rhodoliths and coated grains (Figure 3c), and occasionally among sparse *P. oceanica* bundles as well as within a *Cymodocea nodosa* meadow (Figure 3d). The *Cladocora* bed showed a backscatter response characterized by a texture of bands of high and low signal related to the patchiness of both the coral bed and the rhodolith bed (Figure 2c,d), often mixed and difficult to discriminate one from the other, with areas characterized by higher coral and rhodolith cover identifiable by a higher backscatter. Proceeding deeper, the sandy/detritic bottom was mostly characterized by the abundant presence of *Caulerpa cylindracea* and, occasionally, other macroalgae including *Dudresnaya verticillata* (locally very abundant), *Gracilaria* sp., and *Gongolaria barbata*, without *C. caespitosa* colonies. This area corresponded to a lower backscatter on the acoustic map (Figure 2c,e). Then, reef patches were present on a gently sloping, uniform seabed from 25 to 35 m depth, with their greatest development at 26–29 m depth (Figure 3g). Reef patches were evident from the acoustic map as coherent forms of biogenic origin, elevated from the surrounding sediment (Figure 2e,f), with a weaker acoustic signature compared to coralligenous assemblages, the latter representing hard biogenic substratum mainly produced by the accumulation of coralline red algae and other calcareous organisms (e.g., bryozoans, polychaetes, mollusks, corals) that settle and live one on another in dim light conditions. The *Cladocora* reef covered an overall surface of 13,474 m^2^ (1.35 ha) and was settled on a sandy bottom with gravels where colonies, although large and often coalescing, were unattached (Figure 3e,f). Below 35 m depth, the seabed was mostly characterized by a sandy or detritic bottom, with patchy coralligenous bioconstructions, without the presence of *C. caespitosa* .

### 3.2 *Cladocora* Ground Characterization and Conservation Status

**FIGURE 4:**
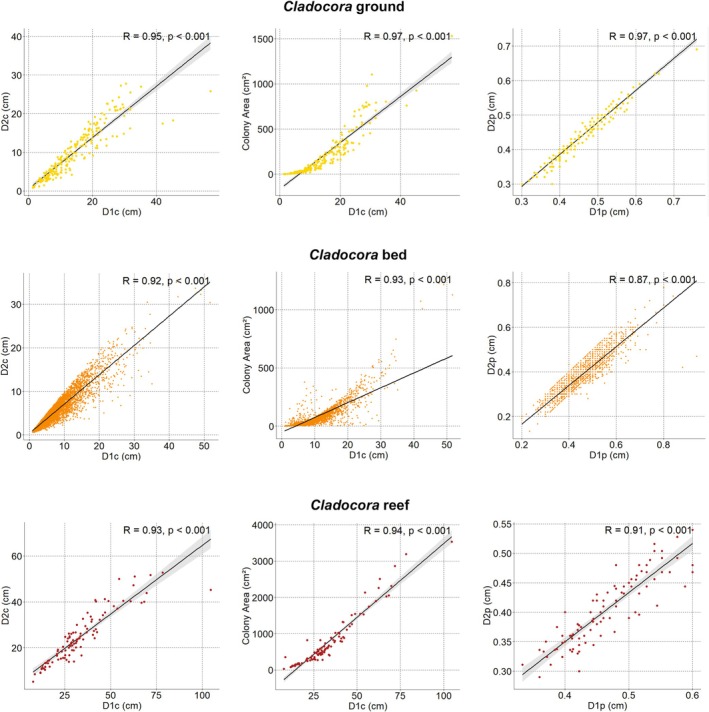
Correlation among morphological metrics of *Cladocora caespitosa* colonies in ground (top), bed (middle), and reef (bottom). Solid black lines represent linear regressions with 95% confidence intervals (gray shading, where visible). Pearson's correlation coefficient (*R*) and the associated *p*‐value are reported for each panel. D1c: Major colony axis; D2c: Minor colony axis; D1p: Major polyp axis; D2p: Minor polyp axis.

**FIGURE 5:**
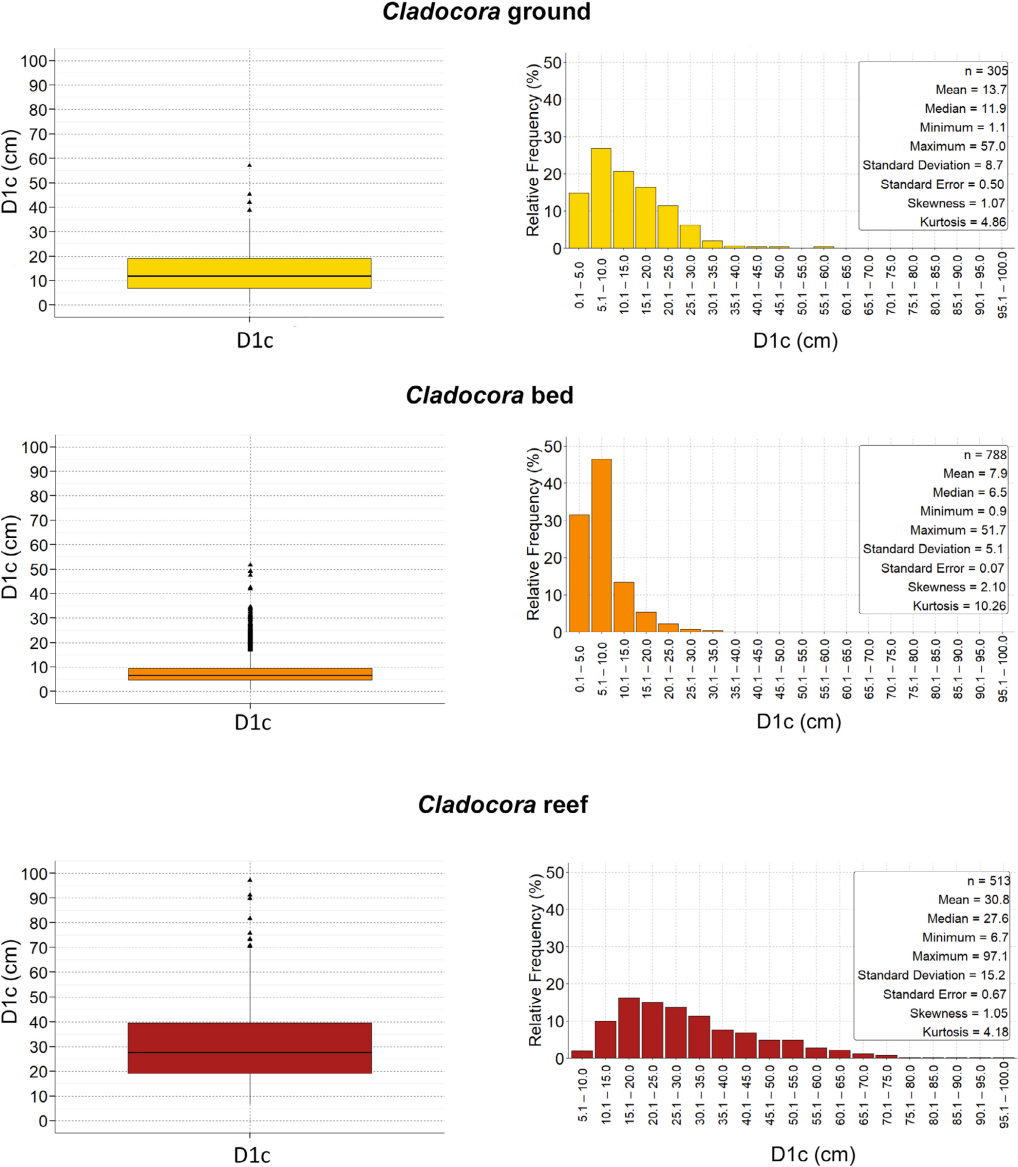
Size structure of *Cladocora caespitosa* colonies in ground (top), bed (middle), and reef (bottom). For each ecosystem type, boxplots (left) and relative frequency histograms (right) show the distribution of colony sizes based on major axis length (D1c). Boxplots show median (horizontal line), 25th and 75th percentiles (boxes), minimum and maximum values smaller than 1.5 times the interquartile range (bars), and outliers (triangles).

A total of 305 *C. caespitosa* colonies were recorded at the *Cladocora* ground, with colony density ranging from 0.54 to 21.01 colonies m^−2^, and a mean density of 4.58 ± 0.45 colonies m^−2^. Colony cover ranged from 0.47% to 34.76%, with a mean of 6.80% ± 0.53%. The distribution of density and cover values showed a significant deviation from the uniform model (K–S test: *D* = 0.69, *p* < 0.001 for density; *D* = 0.59, *p* < 0.001 for cover), indicating marked heterogeneity among sampling units at the scale of the sampling grid. D1c was significantly correlated with both D2c and colony area (Figure 4) and was chosen as the primary size descriptor. D1c ranged from 1.09 to 57.00 cm—corresponding to a colony area of 0.68 and 1530.51 cm^2^, respectively—with most colonies between 7.5 and 20 cm, a median of 11.86 cm, and a mean D1c of 13.67 ± 0.50 cm. The size‐frequency distribution was right‐skewed and leptokurtic, with a peak in the 5.1–15.0 cm classes (Figure 5).

D1p, positively correlated with D2p (Figure 4), ranged from 2.1 to 7.9 mm, with a mean of 4.49 ± 0.01 mm. Both maximum and mean D1p broadly increased with depth, while a correlation was found between D1p and both D1c and colony area (*R* = 0.5, *p* < 0.01 in both cases).

**FIGURE 6:**
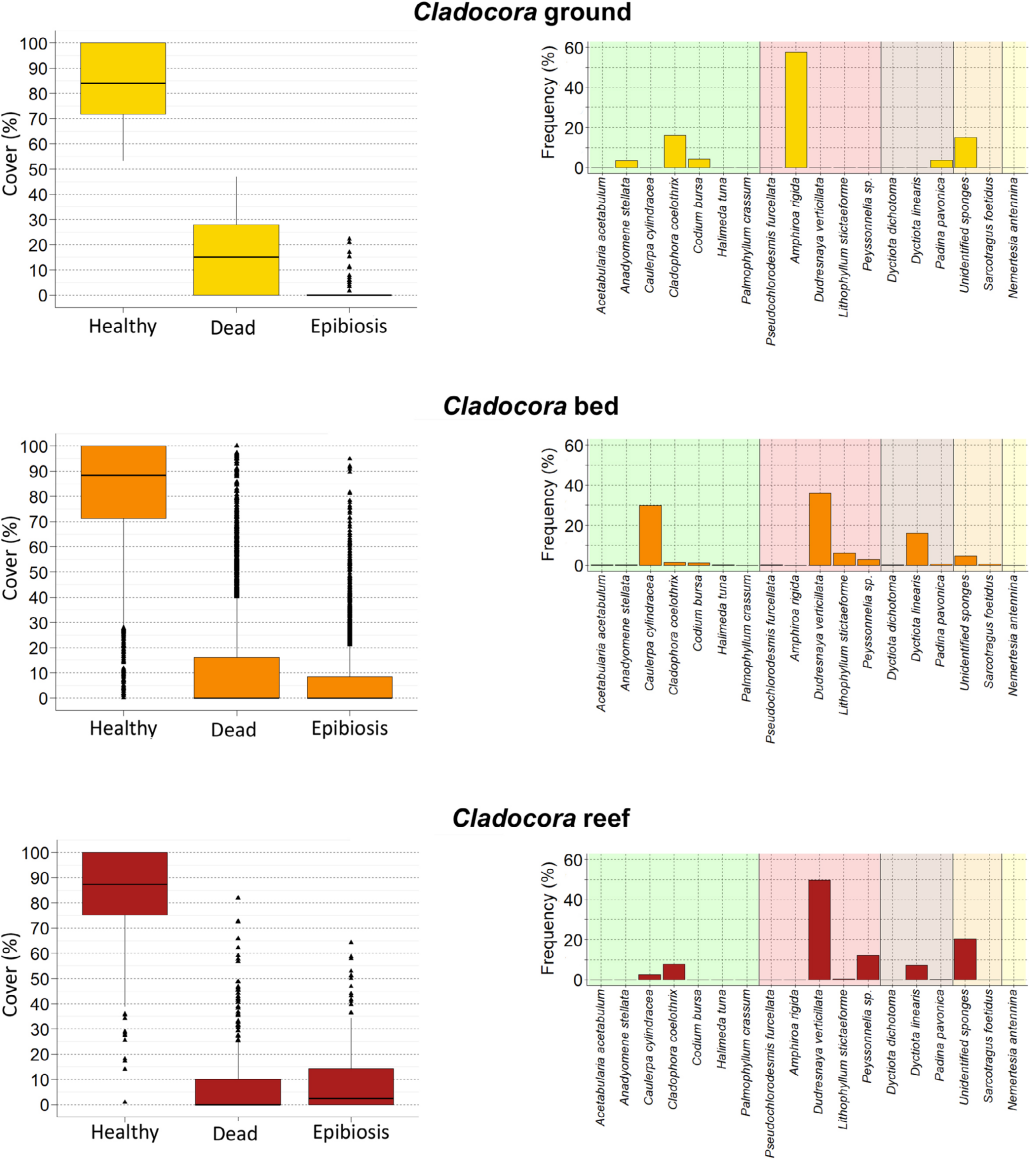
Conservation status and epibiont composition of *Cladocora caespitosa* colonies across ground (top), bed (middle), and reef (bottom). Boxplots on the left display the percentage of healthy surface, mortality, and epibiosis per colony. Bar plots on the right show the frequency of occurrence of epibiont taxa observed over the epibionted colonies.

The population showed a generally healthy conservation status, with 26.60% of the colonies completely healthy, and a further 54% of the population with more than 70% of healthy surface (Figure 6). Broadly, colonies showed a mean healthy surface of 83.40% ± 0.81%. Necrotic surface ranged from 0% to 46.94%, with a mean of 16.05% ± 0.82%.

Epibiosis was found in less than 5% of the colonies and affected 0.49% ± 0.15% of the colony surface, with epibionted surface ranging from 1.69% to 22.10% on affected colonies. More than half of the population (57.65%) was not colonized by any epibiont. The main epibiont was the red alga *Amphiroa rigida* , covering 57.63% of the overall epibionted surface, followed by the green algae *Cladophora coelothrix* (16.11%) and *Codium bursa* (4.21%). Unidentified sponges were detected among the polyps' interstices or on dead coral portions, representing 14.96% of the total epibionted surface. A few other species were occasionally found living on *C. caespitosa* colonies with very low frequency and representing no threat to the colony's health.

**FIGURE 7:**
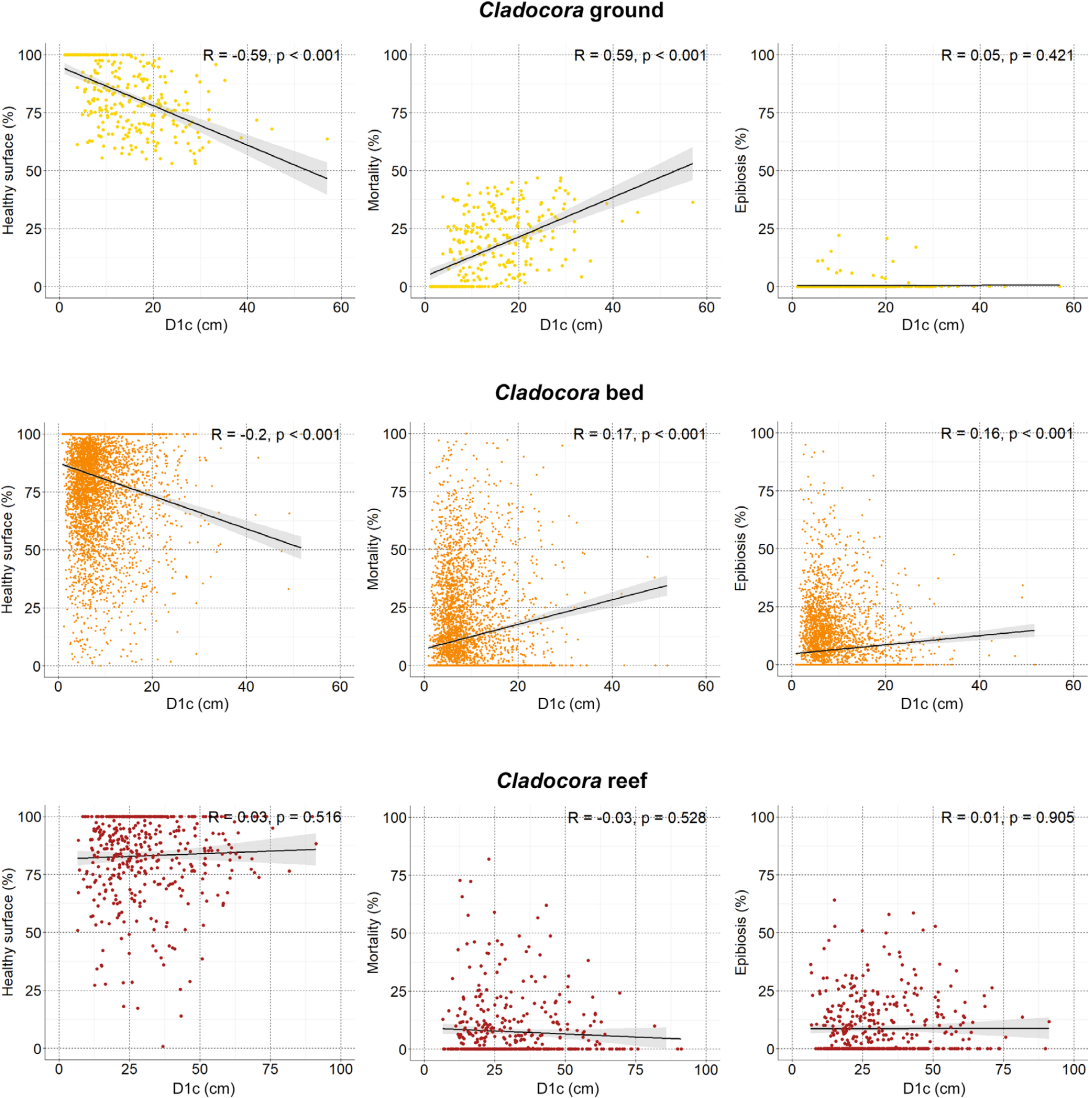
Correlation between colony size (major axis, D1c) and conservation status indicators for *Cladocora caespitosa* within ground (top), bed (middle), and reef (bottom). Solid black lines represent linear regressions with 95% confidence intervals (gray shading). Pearson's correlation coefficient (*R*) and the associated *p*‐value are reported for each panel.

D1c was negatively correlated with the percentage of healthy tissue and positively correlated with necrosis, while its correlation with epibiosis was not significant (Figure 7).

### 3.3 *Cladocora* Bed Characterization and Conservation Status

Based on a total of 788 colonies counted at the *Cladocora* bed, coral density ranged from 1.06 to 26.91 colonies m^−2^, with a mean density of 8.59 ± 1.46 colonies m^−2^. Colony cover ranged between 1.61% and 10.36% of the sea bottom, with a mean of 4.95% ± 0.36%. Density and cover values showed a moderate but significant departure from the uniform model (K–S test: *D* = 0.39, *p* < 0.001 for density; *D* = 0.23, *p* < 0.001 for cover), indicating marked heterogeneity among sampling units at the scale of the sampling grid.

D1c was significantly correlated with both D2c and the colony area (Figure 4) and was chosen as the primary size descriptor. D1c ranged from 0.87 to 51.66 cm (0.55 and 1258.55 cm^2^, respectively), with almost half of the population (46%) falling between 5 and 10 cm D1c, a median of 6.52 cm, and a mean of 7.86 ± 0.12 cm. The size‐frequency distribution was right‐skewed and highly leptokurtic, with a main peak in size classes < 10 cm (Figure 5).

D1p, positively correlated with D2p (Figure 4), ranged from 2.0 to 9.4 mm, with a mean of 4.67 ± 0.01 mm. Both maximum and mean D1*p* broadly increased with depth, while no correlation was found between D1p and both D1c (*R* = 0.001) and colony area (*R* = 0.003).

The *Cladocora* bed showed a generally healthy conservation status, with 35.37% of colonies completely healthy, and a further 42.63% of the colonies with more than 70% of healthy surface (Figure 6). Broadly, colonies showed a mean healthy surface of 82.26% ± 0.29%. Necrotic surface ranged from 0% to 100% (for one colony), with a mean of 11.28% ± 0.26%.

Epibiosis affected 6.20% ± 0.17% of the colony surface, with epibionted surface on affected colonies ranging from 0.56% to 94.87%. Most of the population (64.64%) was not colonized by any epibiont. The main epibionts were *D. verticillata* (36.03% of the overall epibionted surface), *C. cylindracea* (29.93%), and *D. linearis* (16.22%). D1c was negatively correlated with the percentage of healthy tissue and positively correlated with both necrosis and epibiosis (Figure 7).

### 3.4 *Cladocora* Reef Characterization and Conservation Status

**FIGURE 8:**
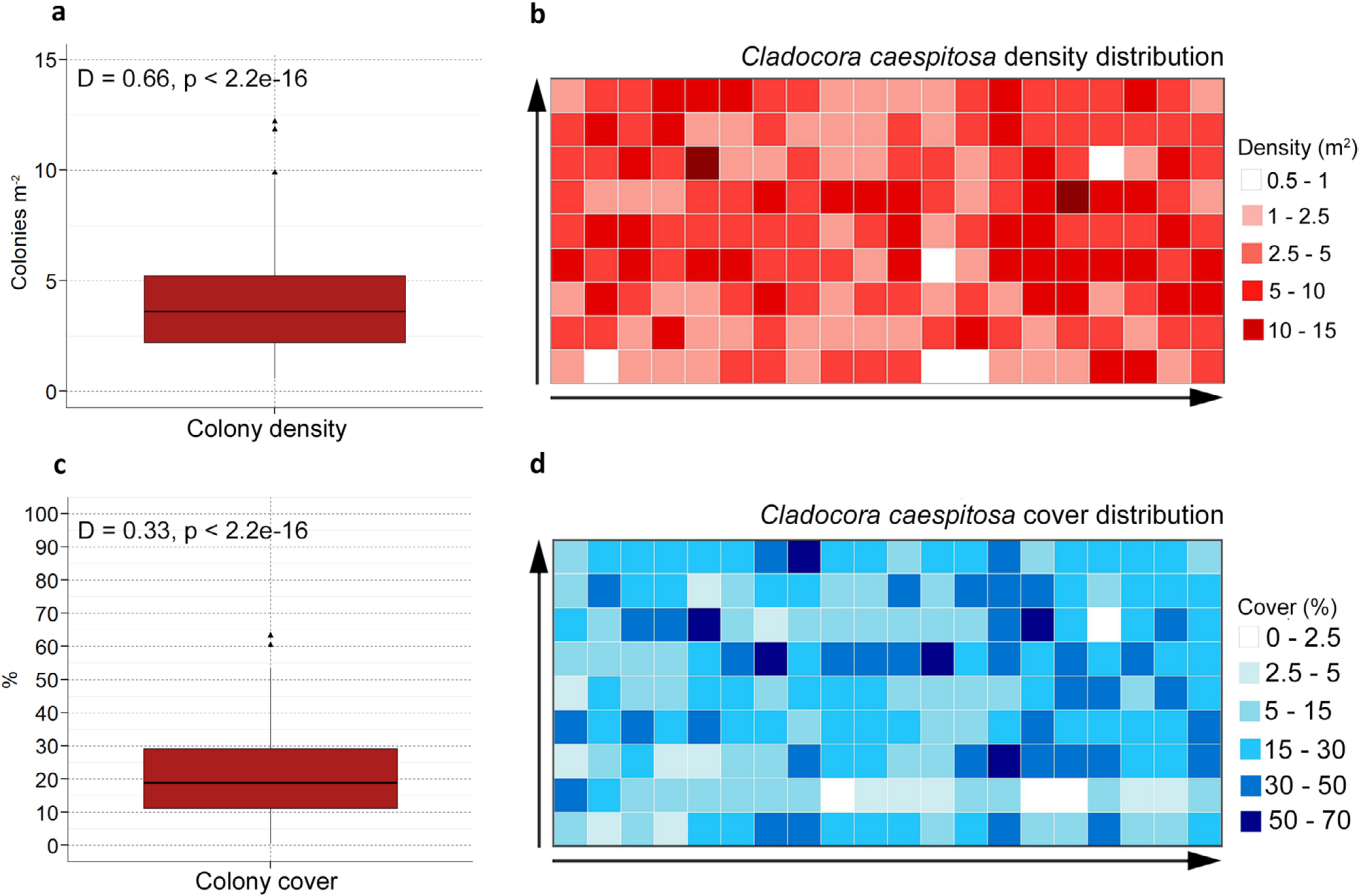
Colonies density and cover of the *Cladocora* reef. Boxplot of (a) density and (c) cover of *Cladocora caespitosa* showing median (horizontal line), 25th and 75th percentiles (boxes), and both minimum and maximum values smaller than 1.5 times the interquartile range (bars); triangles represent outliers. The results of the Kolmogorov–Smirnov non‐parametric test are also shown for each parameter as heatmaps of (b) density and (d) coverage calculated for *C. caespitosa* ; each of the 180 cells represents a different sampling unit.

A total of 513 colonies of *C. caespitosa* were surveyed. Many colonies exhibited coalescence, with 14% (77 colonies) in an advanced coalescence stage so that it was not possible to distinguish the single colonies and they were considered as one in terms of number and morphometric parameters (Figure 3). Colony density ranged from 0.56 to 12.11 colonies m^−2^, although mostly between 2.4–5.1 colonies m^−2^, with a mean density of 3.89 ± 0.17 colonies m^−2^. Colony cover ranged from 0.68% to 63.23%, mostly between 11% and 29%, with a mean cover of 20.79% ± 0.96% along the surveyed reef area. Colony density and cover varied markedly across sampling units, with highly significant deviations from uniformity (Figure 8a,c), showing heterogeneity among sampling units at the scale of the sampling grid (Figure 8b,d). D1c was positively and significantly correlated with both D2c and the colony area (Figure 4), so it was selected as the main descriptor for the colony size. Half of the surveyed population (50%) ranged between 19.17 and 39.51 cm D1c (Figure 5), with a median of 27.58 cm with a mean D1c of 30.81 ± 0.67 cm. The smallest colony measured 6.68 cm D1c (32.17 cm^2^), while the largest one was 97.06 cm D1c (5626.39 cm^2^). The size structure showed a peak corresponding to the size classes between 15.1 and 25.0 cm, with a right‐tailed and leptokurtic distribution (Figure 5).

D1p, positively correlated with D2p (Figure 4), ranged from 3.0 to 8.4 mm, with a mean of 4.83 ± 0.07 mm. Both maximum and mean D1*p* broadly increased with depth, while no correlation was found between D1p and both D1c (*R* = −0.028) and colony area (*R* = −0.040).

The reef was in overall healthy conservation status, with 27.98% of the colonies being 100% healthy and a further 54.52% of the colonies with more than 70% of healthy surface (Figure 6). Colonies' mean healthy surface was 83.77% ± 0.82%. Mortality ranged between 0% and 81.94% of the colony surface, with a mean dead surface of 7.51% ± 0.63% per colony. More than half of the population (54.87%) showed no signs of mortality, and a further 20.13% of the colonies had less than 10% of necrotic surface.

Epibionts covered a mean colony area of 8.73% ± 0.58%, with epibionted colonies affected between 0.58% and 64.16%. Almost half of the population (48.64%) was not epibionted, while 15.98% showed epibiosis on less than 10% of the colony area (Figure 6). Half of the total epibionted surface (49.71%) was due to the red algae *D. verticillata* . A few, unidentified sponges were also abundant, representing 20.22% of the epibionted surface, followed by the red algae *Peyssonnelia* sp. (12.12%) and *C. coelothrix* (7.77%) (Figure 6). Other epibionts were less abundant or very rarely found, and included the green algae *C. cylindracea* and *Halimeda tuna* , the red algae *L. stictaeforme*, and the brown algae *P*. *pavonica*, *D. dichotoma*, and *D. linearis* (Figure 6). In particular, *C. cylindracea* was quite common on the seabed but not overgrowing *C. caespitosa* . Colony health, mortality, and epibiosis were not statistically correlated with D1c (Figure 7).

### 3.5 Comparisons Among *Cladocora* Ground, Bed, and Reef

**FIGURE 9:**
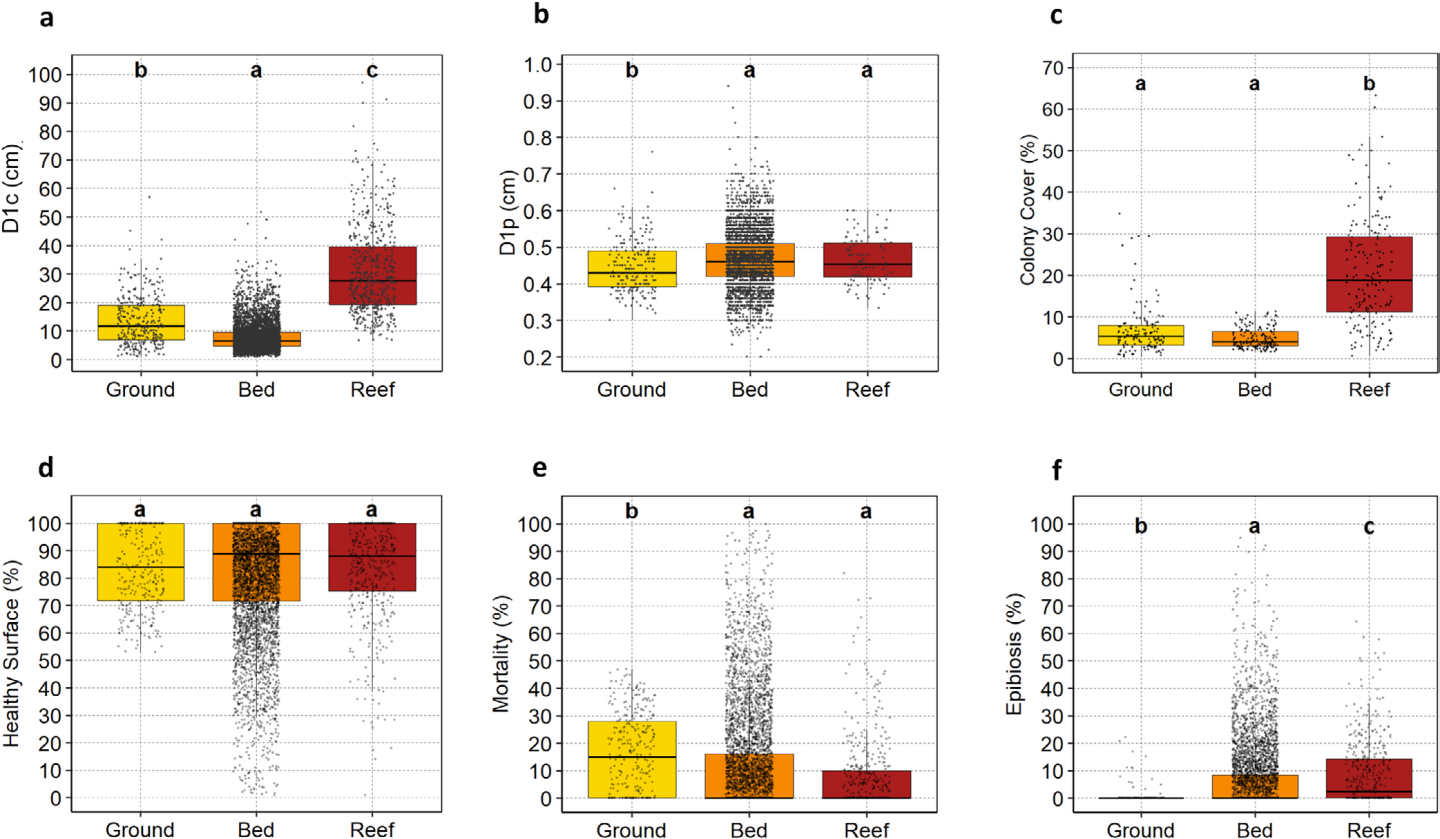
Boxplots showing the comparison of six colony‐level parameters of *Cladocora caespitosa* across the three ecosystem types. (a) Major axis length (D1c), (b) Polyp diameter (D1p), (c) colony cover, (d) healthy surface, (e) mortality (%), (f) epibiosis. Boxes represent interquartile ranges with medians; overlaid points are raw data. Different letters indicate statistically significant differences among habitats (Kruskal‐Wallis followed by Dunn's test with Bonferroni correction, *p* < 0.05).

D1c was significantly different among the three *Cladocora* ecosystems, with the bed having the smaller colonies and the reef the larger ones (Figure 9a). Ground colonies showed smaller polyps compared to both bed and reef ones (Figure 9b). Colony cover was significantly higher in reef colonies compared to both ground and bed ones (Figure 9c), while density was significantly higher in the bed (*p* > 0.001) with respect to both ground and reef. No significant differences were detected in the percentage of healthy surface, which remained consistently high across the three *Cladocora* ecosystem types (Figure 9d). However, mortality was significantly more pronounced in colonies in the ground, while epibiosis was significantly more pronounced in the reef compared to the bed and ground (Figure 9e,f). Thus, among the three ecosystem types, the ground was characterized by intermediate‐size colonies, smaller polyps, higher mortality and lower epibiosis, the bed was characterized by smaller colonies occurring in the form of coralliths, and intermediate levels of epibiosis, while the reef showed larger colonies, higher cover, and higher epibiosis.

### 3.6 Environmental Parameters

**FIGURE 10:**
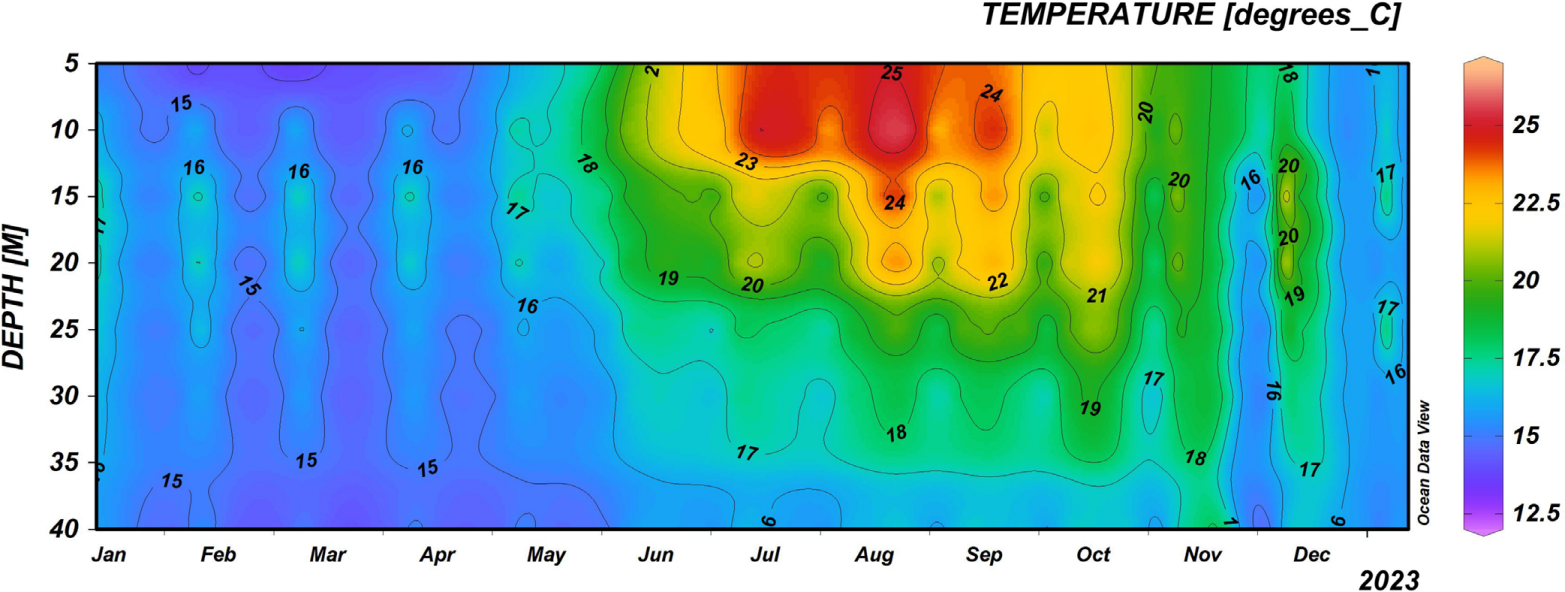
Temperature profile (°C) from 5 to 40 m depth at Tremiti Islands during 2023.

The thermal profile recorded at Tremiti Islands throughout 2023 (Figure 10) showed a pronounced seasonal thermocline, with shallow waters exceeding 25°C during summer (July–August). The *Cladocora* ground (above 10 m depth) was exposed to temperatures > 24°C for approximately 3 months. Such high temperatures were also recorded up to 23 m during the summer peak (August), thus also affecting the *Cladocora* bed. On the contrary, the *Cladocora* reef was exposed to temperatures between 15°C and 20°C throughout the year (Figure 10). A progressive water cooling below 20°C occurred from October onward in both shallow and deeper zones.

### 3.7 Spatial Distribution of *Cladocora caespitosa* Ecosystems in the Mediterranean Sea

**FIGURE 11:**
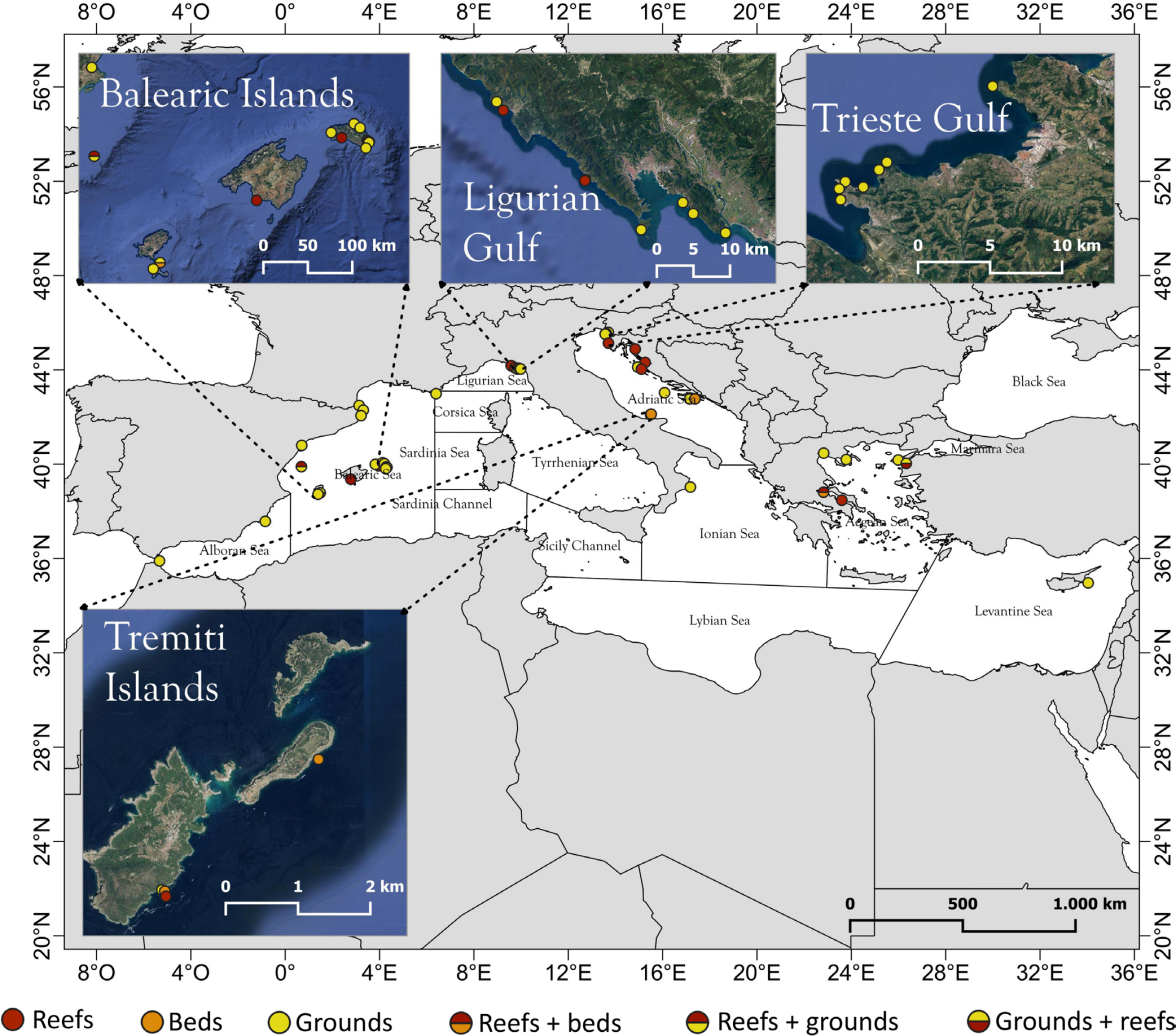
Map of the known distribution of *Cladocora caespitosa* ecosystems, with indication of grounds, beds, reefs, and occurrence of mosaics. A focus on areas with numerous records is provided.

The *Cladocora* ecosystems currently reported in the Mediterranean Sea, including those reported in this study, sum to 40 grounds, 3 beds, and 13 reefs (Figure 11 and Table 1). The three ecosystem types are not always isolated from one another, sometimes occurring as a continuum of different formations that can be stratified with depth or simply mixed.

**TABLE 1:** Living *Cladocora caespitosa* formations recorded in the Mediterranean Sea. The different ecosystem types are indicated (B, Bed; G, Ground; R, Reef); when one main type and a secondary one are present, the latter is indicated in brackets. For colonies density and cover, both mean and maximum values are reported.

Basin	Country	Location	Site	Depth (m)	Ecosystem type	Extension (m^2^)	Density (colonies m^−2^)	Cover %	References
Alboran Sea	Spain	Ceuta	Bay of Benzú	20	G				Ocaña et al. (2009)
		Murcia	Bay of Portman	5–11	G	500	~0.4		J Templado (2007) personal observation in Chefaoui et al. (2017)
Balearic Sea	Spain	Formentera	S'Espardelló Islet	5–14	G (+B)			18.96; up to 35.5	Kersting et al. (2017a); Kersting et al. (2017b); J Templado (2007) personal observation in Chefaoui et al. (2017)
			Es Banc	8–12	G	387.5		33.7	Pons‐Fita et al. (2020)
		Mallorca	Capo Blanco	36	R				OCEANA (2006); R Aguilar, personal communication in Casado‐Amezúa et al. (2015)
		Menorca	Cala Galdana	5–10	R			1.43; up to 6.34	JA Fayos (2014), personal communication in Casado‐Amezúa et al. (2015); Kersting et al. (2023)
			Ciutadella	4–12.5	G			2.51; up to 9.75	Kersting et al. (2023)
			Fornells	4–14.5	G			5.04; up to 12.4	Kersting et al. (2023)
			Na Macaret	1.5–12	G			1.27; up to 3.3	J Templado (2007) personal observation in Chefaoui et al. (2017); Kersting et al. (2023)
			Illa del Rei and Fonduco	1.5–4	G			~4.3; up to 4.63	Kersting et al. 2023
			Pedrera	4–11	G			4.52; up to 9.60	Kersting et al. (2023)
			La Mola	1.5–18	G			2.06; up to 6.14	Kersting et al. (2023)
			Cala San Esteve	4–13.5	G			1.75; up to 3.50	J Templado (2007) personal observation in Chefaoui et al. (2017); Kersting et al. (2023)
			Biniancolla	1.5–5.5	G			2	Kersting et al. (2023)
		Columbretes Islands	Illa Grossa Bay	5–27	R (+G)	1150; 2900^a^	up to 5.5	1.9; up to 80	Kersting and Linares (2012)
		Tarragona	L'Ampolla	3–6	G				Movilla et al. (2012)
		Cap de Creus	Messina Islet	26–28	G				Quintano et al. (2025)
		Montgrí, Medes and Baix Ter	La Calella	19–22	G				Quintano et al. (2025)
	France	Banyuls‐sur‐mer			G				Romans P. (2014), personal communication. in Casado‐Amezúa et al. (2015)
		Hyères Islands	Port‐Cros Bay	2	G	500	1–2		Laborel and Laborel‐Deguen (1978)
Ligurian Sea	Italy	La Spezia	Bonassola	7–10	G				Peirano (2007); Ferrier‐Pagès et al. (2011)
			Bonassola	27–29	R				Morri et al. (1994); Peirano et al. (1994); Peirano (2007)
		Riomaggiore	Capo di Montenero	10	G				Peirano et al. (1994)
				26–27	R				Morri et al. (1994); Peirano et al. (1994); Peirano (2007)
		Palmaria Island		20	G				Peirano et al. (1994)
		Gulf of La Spezia	Lerici	8–10	G				Ferrier‐Pagès et al. (2011)
			Fiascherino Bay	8–10	G				Peirano et al. (2001, 2005)
		Bocca di Magra	Punta Bianca	3–10	G		up to 8		Peirano et al. (1994, 1999, 2001); Peirano (2007); Ferrier‐Pagès et al. (2011); Azzola et al. (2022)
Ionian Sea	Italy	Crotone	Capo Donato	4–10	G	8000^a^			Lumare (1966)
Adriatic Sea	Italy	Tremiti Islands	San Domino	5–10	G	25,687	4.6; up to 21.0	6.80; up to 34.76	This study
				15–22	B	11,987	8.6; up to 26.9	4.95; up to 10.36	Chimienti et al. (2025); this study
				25–30	R	14,364	3.9; up to 12.1	20.79; up to 63.23	This study
			San Nicola	15–23	B	35,114	5.5; up to 15.1	5.98; up to 10.04	Chimienti et al. (2025); this study
	Slovenia	Gulf of Trieste	Debeli rtič	3–6	G		0.8; up to 1		Zunino et al. (2018)
			Cape Ronek	8, 12–21	G	20,000^a^	6.5; up to 8.1		Lipej et al. (2016); Pitacco et al. (2014); Zunino et al. (2018)
			Strunjan	4–5	G		1.6; up to 1.8		Zunino et al. (2018)
			Pacug	6	G		2.2		Zunino et al. (2018)
			Cape Madona	8–10	G		0.5		Zunino et al. (2018)
			Piran	4–8	G		1.8; up to 3.4		Zunino et al. (2018)
			Bernardin	5	G		3.6	Up to 31	Schiller (1993); Zunino et al. (2018)
	Croatia	Lim Channel		19	R				Abel (1959); Kružić et al. (2012)
		Prvic Island		11–19	R	190			Kružić and Benković (2008)
		Pag Island		15–21	R	120			Kružić and Benković (2008)
		Dugi Otok Island			G				Kružić (2005)
		Iz Island		12–16	R	150			Kružić and Požar‐Domac (2007)
		Vis Island	Komiža Bay		G				Kružić (2005)
		Glavat Islet			G				Kružić (2005)
		Mljet National Park	Veliko Jezero	6–18	R (+G)	650			Kružić and Požar‐Domac (2003); Kružić and Benković (2008); Kružić et al. (2025)
				Below 18 m	B				Kružić et al. (2025)
Aegean Sea	Greece	Gulf of Atalanta	Kako‐Kephali	5–7	R				Laborel (1961)
			Strongylo Islet	5–18	R (+B)			Up to 100	Laborel (1961)
		Diaporos Island	Vourvourou	15–20	G			2	Koukouras et al. (1998)
		Thermaikos Gulf	Nea Michaniona	3–15	G				Ganias (2025)
	Turkey	Gökçeada Island		3–15	G		0.1	0.27	Zibrowius (1979); Güresen et al. (2015)
		Dardanells	Çanakkale Strait	4–5	G (+R)	720		Up to ~5	Özalp and Alparslan (2016); Özalp and Casado‐Amezúa (2024)
Levantine Sea	Cyprus	Krio Nero		2–10	G				Jiménez et al. (2016)

^a^ Overall cumulative area interested in the *Cladocora* ecosystem.

Grounds are the most common throughout the basin and might occur on bare rocky substrate (barren), among macroalgae associations, as well as among seagrass meadows, broadly from 2 to 20 m depth. They can also occur as a mosaic of ground and reef (i.e., Columbretes Islands, Spain; Mljet National Park, Croatia; Dardanells, Turkey), or ground and bed (i.e., Formentera, Spain).

Beds are particularly rare. The most relevant found so far are in Mljet, below the main reef at 18 m depth, and at the Tremiti Islands as an extensive ecosystem mostly between 15 and 22 m depth. Additional beds have been observed off Formentera (Spain) and the Gulf of Atalanta (Greece) as a minor component of a ground (5–14 m) and a reef (5–18 m), respectively.

Reefs are also rare, some of them being reported more than 20 years ago and never monitored afterward. They can occur on rocky bottoms (i.e., off Croatia, Greece, and Spain), coralligenous habitat (i.e., Ligurian coast), or on sandy bottom (i.e., Tremiti Islands), from 4 to 30 m depth depending on the local conditions.

## 4 Discussion

### 4.1 The *Cladocora* Ecosystems

The environmental setting within the Tremiti Islands Marine Protected Area provided an unprecedented opportunity to characterize and distinguish the three different ecosystem types that *C. caespitosa* can form. Ground, bed and reef showed different distribution and morpho‐ecological features. Their coexistence within the Tremiti Islands supports the idea that local environmental conditions shape the development and persistence of these biogenic formations, which may be separated both spatially and bathymetrically, or may occur as a continuum. For instance, in one of the first descriptions of the habitat‐forming capabilities of *C. caespitosa* , Laborel (1961) reported a stratification of the *Cladocora* ecosystems from the Gulf of Atalanta (Greece) with a rocky bottom dominated by *C. caespitosa* at 5–9 m depth, a “plateaux à Cladocora libres” at 9–11 m depth, and a “dalle concrétionnée bordée par un véritable bourrelet frangeant” from 11 to 18 m depth. More than 60 years later, we report a similar situation in the Tremiti Islands, where, however, both bed and reef occur deeper than those off Greece, and the three *Cladocora* ecosystems are not strictly adjacent, their separation being marked by portions of the seabed without coral colonies. On the contrary, Kružić et al. (2025) showed that the *Cladocora* bed at Mljet National Park (Croatia) occurs deeper than the reef and originates from its fragmentation, while Kersting et al. (2017b) highlighted that coralliths from Formentera (Spain) represent about 10% of the population, possibly originating from the above *Cladocora* ground.

The marked bathymetric zonation of the three *Cladocora* ecosystems is possibly linked to a combination of biotic and abiotic drivers that vary with depth. A *Cladocora* ground above 10 m suggests that the corals are somehow adapted to high temperature fluctuations and heatwave occurrence, as well as favored by the action of the waves that possibly undermine algal dominance by breaking thalli, thus reducing competition. This was particularly evident on barren rocks, where the grazing activity by sea urchins further reduces algal competition without affecting *C. caespitosa* . Deeper rocky substrates were almost devoid of *C. caespitosa* , which dominated the seabed again between 15 and 23 m depth, but this time in the form of coralliths on a soft bottom. Here, bottom currents support the formation and the maintenance of both coralliths and rhodoliths, whose almost complete absence above 15 m is possibly linked to higher thermal stress and stronger hydrodynamic conditions (Chimienti, Rizzo, et al. 2020; Chimienti et al. 2025). The isolated presence of *Cladocora* reefs between 25 and 35 m depth might have originated from the rollover of coralliths down to these depths in the past, where colony growth has continued over time, supported by more stable conditions that allowed for colony expansion and habitat accretion. In fact, colonies with a large enough size not to be pushed by bottom currents and grow in one direction are also present on the *Cladocora* bed, although with a maximum D1c much lower than that found on the reef (52 vs. 97 cm). Although the associations between abiotic factors and ecosystem type are consistent with previous studies and provide plausible ecological explanations, it is important to note that they are observational and not necessarily causal.

The Tremiti ground stands out as the largest and most continuous recorded so far globally. However, although grounds have been commonly reported across the basin, they remain largely unmapped since large‐scale mapping techniques are not effective in distinguishing the *Cladocora* ground from other infralittoral rocky assemblages. Thus, grounds with comparable extensions to those of the Tremiti Islands are likely to exist in other Mediterranean areas, such as the Ligurian and the eastern Adriatic coasts (e.g., Peirano et al. 2001; Lipej et al. 2016). On the contrary, beds have been rarely found in the basin, the Tremiti one representing the only one documented so far as a distinct ecosystem. Reefs remain rare and often poorly characterized, the occurrence of living reefs being much less common than fossil ones (Aguirre and Jiménez 1998; Peirano et al. 2009; Ingrosso et al. 2018). The Tremiti reef is among the largest recorded so far, comparable to the most extensive reefs such as those at Columbretes Islands (Kersting and Linares 2012) and at Mljet National Park (Kružić and Benković 2008). These ecosystems, possibly remnants of a broader historical distribution, may represent key ecological and evolutionary refugia. Further unchecked *Cladocora* ecosystems are likely to occur within the basin, including off the coast of North Africa. For instance, extensive formations might be present off Tunisia, where fossil formations are known (Zibrowius 1980), while it is unclear whether living colonies are still present.

### 4.2 Sampling Biases and Sampling Units

The overall distribution of the *Cladocora* ecosystems at the basin scale highlighted a bias in exploration and sampling effort along the coast of North Africa and the Eastern Mediterranean Sea. However, a further bias might occur when comparing literature data, since a shared methodology for surveying *Cladocora* ecosystems is lacking. Density assessment of *C. caespitosa* colonies has been extensively carried out using direct visual census within different techniques with different levels of precision (e.g., taped transects, defined quadrats, towed visual census, visual estimation of a sampling area) and using a wide range of sampling units, encompassing 1 m^2^ (Peirano et al. 2001), 5 m^2^ (Kersting and Linares 2012), 12.6 m^2^ (circle with 2 m radius; Güresen et al. 2015), 25 m^2^ (5 × 5 m; Azzola et al. 2022), ~15–30 m^2^ (various transects from 15 to 30 m long; Zunino et al. 2018), ~50 m^2^ (50 × 1 m; Özalp and Alparslan 2016), and 150 m^2^ (50 × 3 m; Chimienti et al. 2025). Similarly, cover assessment has always been carried out visually on sampling units of 0.2 m^2^ (20 × 0.01 m; Kersting et al. 2017b), 0.5 m^2^ (50 × 0.01 m; Kersting et al. 2023; Pons‐Fita et al. 2020), ~50 m^2^ (50 × 1 m; Kersting and Linares 2012; Özalp and Alparslan 2016), and 150 m^2^ (50 × 3 m; Chimienti et al. 2025). In other cases, population parameters have been collected through random visual estimations or in other unclarified ways. This aspect must be considered when monitoring benthic ecosystems, since the size of the sampling unit and the technique used can influence the output. Larger sampling units tend to integrate broader seafloor heterogeneity and typically yield lower or more averaged density and cover values, as they encompass both areas rich in colonies and adjacent less dense patches. In contrast, smaller units provide higher resolution and can capture fine‐scale variability, potentially inflating density or cover estimates when small hotspots fall within the sampled frame. Similarly, the ability to detect small colonies—and thus accurately represent the left tail of the size‐frequency distribution—declines markedly when using large units or rapid visual census techniques. As a result, differences in sampling unit and survey method can generate systematic biases that complicate comparisons across studies or sites unless methodologies are standardized. For instance, the *Cladocora* bed at *Grotta del Sale* (Tremiti Islands), monitored in this study with a sampling unit of 0.8 m^2^, has been previously described based on 50 × 3 m transects representing one large sampling unit of 150 m^2^, corresponding to transect GDS3 in Chimienti et al. (2025). Being monitored within the same year and excluding large population fluctuations, density resulted slightly higher with the small sampling unit (8.59 ± 1.46 vs. 7 colonies m^−2^), while cover resulted markedly higher (4.95% ± 0.36% vs. 2.91%). The choice of the sampling unit represents a compromise between avoiding subjectivity in the sampling—the larger the sampling unit, the more inclusive the sampling—and maximizing the resolution, particularly when using photographic surveys. Based on species–area curves, Weinberg (1978) proposed a minimal area of 2 m^2^ for the sampling of sessile invertebrates—mainly Octocorallia—in the Mediterranean coralligenous habitat, later adopted in several studies based on visual surveys on coral communities (e.g., Ambroso et al. 2014; Chimienti, De Padova, et al. 2020; Chimienti et al. 2023). However, such a sampling unit is not effective in the photographic survey of *C. caespitosa* ecosystems, particularly in the presence of many small colonies, like in corallith beds, or in the case of poor (< 1 m) visibility. The adoption of a 0.8 m^2^ sampling unit in this study guaranteed high photographic resolution to identify even the smaller colonies and to collect a suite of data at the population level. The establishment of a consensus in the monitoring protocol of *C. caespitosa* will be essential in the future to allow the appropriate comparison of populations monitored by different research teams. In this context, image analysis is still scantly used although proving to be a robust, objective, and replicable way to monitor coral ecosystems, also reducing the operational time underwater compared to direct measurements by divers.

### 4.3 Ecology and Morphometry

The differences in colony density and cover among the three *Cladocora* ecosystems at the Tremiti Islands reflect distinct population dynamics and structural roles. The ground showed a high mean density with a relatively low cover related to the presence of many small to medium‐sized colonies (5–20 cm) that are still spatially isolated. Both density and cover are among the highest known for *Cladocora* grounds, together with those found along the coast of Formentera (Kersting et al. 2017a; Chefaoui et al. 2017; Pons‐Fita et al. 2020). The *Cladocora* bed showed the highest density in the study area, possibly linked to fragmentation due to rollover, and low cover related to the small size of most of the colonies, without data from other sites for comparison. The reef displayed lower colony density compared to both ground and bed, and markedly higher cover, reflecting the dominance of large colonies with coalescing processes sometimes leading to a density underestimation. Recorded density is comparable to the only other *Cladocora* reef quantified in terms of colony density, such as that of the Columbretes Islands (Kersting and Linares 2012). The mean cover is remarkably higher than that recorded in the reefs of both Menorca (Kersting et al. 2023) and the Columbretes Islands (Kersting and Linares 2012). The maximum cover is in line with the few other living reefs known so far, where maximum cover tends to be high due to the increased density and coalescence processes, independent of the sampling units used. The Tremiti reef also displayed a pronounced heterogeneity in colony density and cover among sampling units, consistent with the mosaic‐like distribution observed at the Columbretes Islands (Kersting and Linares 2012). This heterogeneity may reflect the inherently low dispersal capacity of *C. caespitosa* eggs and larvae (Kružić et al. 2007; Casado‐Amezúa et al. 2014), which can favor localized concentration of colonies over time.

The differences in terms of size structure across the three *Cladocora* ecosystems at the Tremiti Islands highlighted distinct demographic patterns. The moderately right‐skewed and leptokurtic distribution of the *Cladocora* ground, consistent with that reported for other ground‐forming populations shallower than 10–15 m (i.e., Peirano et al. 2001; Zunino et al. 2018; Azzola et al. 2022; Kersting et al. 2023), may reflect intermittent recruitment and moderate survival over time of most of the colonies. On the contrary, grounds deeper than 25 m off the Spanish coast have shown the dominance of larger size classes, between 20 and 40 cm D1c (Quintano et al. 2025). The *Cladocora* bed exhibited a markedly right‐skewed and leptokurtic distribution, with most colonies falling within the smallest size class (0.1–5.0 cm) and a highly uneven size structure, as also found off Formentera (Kersting et al. 2017b). Such a pattern may be consistent with the dynamic nature of corallith beds, where frequent turnover, fragmentation, and environmental stressors limit the ability of colonies to reach larger sizes. In strong contrast, the *Cladocora* reef displayed the largest colony sizes, with a size‐structure reflecting a mature population under prolonged stability, successful long‐term growth, and potentially low disturbance levels triggering advanced coalescence and accretional potential. A similar size‐frequency distribution was found at the Columbretes Islands (Kersting and Linares 2012), where, however, the dominance of the 10–30 cm size classes was more evident than in the Tremiti Islands' population. Such marked dominance of intermediate size classes might be linked to the shallower occurrence of the Columbretes reef (mostly 10–20 m depth, although the complete depth range is 5–27 m), compared to the Tremiti reef (26–35 m). This pattern suggests that shallower populations are more subject to environmental fluctuations detectable in a more skewed and leptokurtic structure, regardless of the type of ecosystem formed. For instance, the reef occurring shallower than 10 m depth at Cala Galdana, Menorca, is characterized by colonies of around 10 cm D1c, although with the presence of some large colonies (Kersting et al. 2023), while the grounds at Cap de Creus and Montgrì, occurring at 26–28 and 19–22 m depth, respectively, showed the abundance of larger size classes (Quintano et al. 2025). Thus, environmental stressors varying locally with depth (e.g., hydrodynamic forces and wave action, temperature fluctuations, heatwaves), combined with edaphic characteristics, nutrients regime (*sensu* Quintano et al. 2025), and biological interactions, influence the population structure and the type of ecosystem formed by *C. caespitosa* , where grounds, beds and reefs might also represent different responses toward environmental forces. Such ecological plasticity is already known in tropical scleractinian corals (e.g., Todd 2008 and references therein), mediated by reproductive processes and symbiosis (Hoogenboom et al. 2008). Furthermore, the three different ecosystem types might be sort of ecological successions in which *C. caespitosa* initially forms grounds and/or beds that eventually become reefs under stable conditions.

### 4.4 Conservation Implications

The three *Cladocora* formations appear to represent a gradient of demographic maturity and ecological stability, from recruitment‐dominated grounds and beds to mature, framework‐building reefs. The ability of *C. caespitosa* to persist and thrive under various conditions highlights the importance of targeted conservation strategies. Protecting the full spectrum of the *Cladocora* ecosystems will be key to safeguarding the ecological roles and evolutionary potential of this emblematic Mediterranean coral, particularly given the rarity of both beds and reefs at the basin scale. In fact, conservation actions impairing local impacts might increase the ability of these ecosystems to cope with large‐scale and global stressors. In the case of the Tremiti Islands, *C. caespitosa* colonies across all ecosystem types showed high percentages of healthy surface and low mortality levels, with no fully necrotic or bleached colonies recorded. These results are notable given the increasing frequency of marine heatwaves in the Mediterranean Sea, driving bleaching and mortality events (Kersting et al. 2013; Garrabou et al. 2022; Kružić et al. 2012, 2025). During the monitored year, seawater temperature did not reach the 28°C threshold that is known to trigger bleaching and consequent mortality in *C. caespitosa* according to Rodolfo‐Metalpa et al. (2005). These authors also showed that a prolonged exposure (i.e., 5 weeks) to 24°C causes stress and tissue necrosis in half of the monitored *C. caespitosa* nubbins. Interestingly, despite ground colonies being exposed to temperatures above 25°C for several consecutive months at the Tremiti Islands, they maintained a high percentage of healthy tissue, suggesting a potential local adaptation or acclimatization. However, mortality was significantly higher in the ground and lower in the reef, with intermediate values in the bed, while epibiosis showed an opposite trend, being higher in the reef and lower in the ground. These patterns might be linked to the increasing environmental stability from shallow to deep, including the reduction of thermal fluctuations and the gradual protection against warm temperature exposure. Furthermore, the lack of overgrowth by invasive species like *C. cylindracea* and *D. verticillata* —common on the seabed but not over the colonies—agrees with the hypothesis that *C. caespitosa* has allelochemical defenses (Kersting et al. 2014).

Differences in the relationship between colony size and conservation indicators across the three *Cladocora* ecosystem types reflect both intrinsic biological dynamics and specific environmental conditions. In the *Cladocora* ground, the strong negative correlation between D1c and healthy surface, alongside the strong positive correlation with mortality—as also observed in other areas (Rodolfo‐Metalpa et al. 2005; Azzola et al. 2022; Quintano et al. 2025), suggests a clear size‐dependent decline in colony health: larger colonies in *Cladocora* ground are generally older and more exposed to cumulative stressors. On the contrary, the absence of a significant correlation between D1c and epibiosis suggests that epibiont colonization is not necessarily related to colony size within the *Cladocora* ground, but it is possibly influenced by local conditions impeding epibionts' survival (e.g., thermal variability and hydrodynamism), as well as by biological interactions (e.g., grazing) removing both competitors and epibionts over dead coral portions and around the colonies. The correlation between D1c and both healthy surface and mortality was maintained in the *Cladocora* bed, while D1c and epibiosis were positively correlated, suggesting that, as coralliths stabilize and grow, they present a larger surface area for opportunistic colonization. Conversely, the reef showed no significant correlation between colony size and any conservation indicator, possibly linked to a reduced environmental stress for the corals that allows them to remain healthy. However, recurrent coalescence processes may affect these correlations, with larger colonies not necessarily being older. Especially in *Cladocora* reefs, coalesced frameworks can produce large morphological units that result from the fusion of multiple colonies, meaning that colony size does not always represent colony age or time since recruitment.

Despite the overall healthy conservation status, *Cladocora* reefs might be more vulnerable to climate change, particularly to global warming, since these populations have not been previously exposed to warm temperatures and heatwaves that can quickly impair their healthy conditions, as recently happened to other Mediterranean coral ecosystems at similar depths (e.g., Coma et al. 2009; Chimienti et al. 2021, 2023; Garrabou et al. 2022). From a conservation perspective, the spatial resolution of our imagery limits the reconstruction of true 2D colony arrangements, preventing a full understanding of how colonies interact and expand across *Cladocora* frameworks. Incorporating georeferenced colony positions in future monitoring would enable the application of dedicated spatial analyses. Such approaches could identify zones of localized recruitment, potential source/sink areas, and spatial pathways of colony expansion, providing key elements for prioritizing protection and designing effective restoration strategies. Considering its vulnerability to both global and local anthropogenic threats, further monitoring of the Tremiti reef over time could support an informed management of human activities and targeted conservation measures for this rare ecosystem within the Tremiti Islands MPA and the whole Mediterranean Sea.

## Author Contributions

**Giovanni Chimienti:** conceptualization (lead), data curation (equal), formal analysis (equal), funding acquisition (lead), investigation (lead), methodology (lead), project administration (lead), resources (equal), supervision (lead), visualization (equal), writing – original draft (lead), writing – review and editing (lead). **Andrea Tursi:** data curation (equal), formal analysis (lead), investigation (equal), methodology (equal), validation (equal), writing – original draft (equal), writing – review and editing (equal). **Alessia Logrieco:** data curation (equal), investigation (equal), writing – review and editing (equal). **Francesco De Giosa:** investigation (equal), writing – review and editing (equal). **Francesco Mastrototaro:** investigation (equal), resources (equal), supervision (equal), writing – review and editing (equal).

## Funding

This work was supported by the European Regional Development Fund, POR‐POC Puglia 2014‐2020 – Asse VI Azione 6.5 sub.

## Conflicts of Interest

The authors declare no conflicts of interest.

## Supporting information


**Appendix S1:** ece372871‐sup‐0001‐AppendixS1.zip.

## Data Availability

Data used in this study is available in the main text and as Supporting Information of this paper. Additionally, data about the Cladocora bed are already published at https://doi.org/10.1038/s41598‐025‐01554‐6.
